# Roll tilt self-motion direction discrimination training: First evidence for perceptual learning

**DOI:** 10.3758/s13414-019-01967-2

**Published:** 2020-01-02

**Authors:** Manuel P. Klaus, C. G. Schöne, M. Hartmann, D. M. Merfeld, M. C. Schubert, F. W. Mast

**Affiliations:** 1 Department of Psychology, University of Bern, Fabrikstrasse 8, 3012 Bern, Switzerland; 2 Department of Otorhinolaryngology, Head and Neck Surgery, University Hospital Bern, University of Bern, Bern, Switzerland; 3 Faculty of Psychology, Swiss Distance University Institute, Brig, Switzerland; 4 The Ohio State University, Columbus, OH USA; 5 Laboratory of Vestibular Neuro Adaptation, Department of Otolaryngology - Head and Neck Surgery, Johns Hopkins University, Baltimore, MD USA; 6 Department of Physical Medicine and Rehabilitation, Johns Hopkins University, Baltimore, MD USA

**Keywords:** Vestibular System, Self-Motion Perception, Roll Tilt, Perceptual Learning, Multisensory Processing

## Abstract

Perceptual learning, the ability to improve the sensitivity of sensory perception through training, has been shown to exist in all sensory systems but the vestibular system. A previous study found no improvement of passive self-motion thresholds in the dark after intense direction discrimination training of either yaw rotations (stimulating semicircular canals) or y-translation (stimulating otoliths). The goal of the present study was to investigate whether perceptual learning of self-motion in the dark would occur when there is a simultaneous otolith and semicircular canal input, as is the case with roll tilt motion stimuli. Blindfolded subjects (n = 10) trained on a direction discrimination task with 0.2-Hz roll tilt motion stimuli (9 h of training, 1,800 trials). Before and after training, motion thresholds were measured in the dark for the trained motion and for three transfer conditions. We found that roll tilt sensitivity in the 0.2-Hz roll tilt condition was increased (i.e., thresholds decreased) after training but not for controls who were not exposed to training. This is the first demonstration of perceptual learning of passive self-motion direction discrimination in the dark. The results have potential therapeutic relevance as 0.2-Hz roll thresholds have been associated with poor performance on a clinical balance test that has been linked to more than a fivefold increase in falls.

**Electronic supplementary material:**

The online version of this article (10.3758/s13414-019-01967-2) contains supplementary material, which is available to authorized users.

## Introduction

Perceptual learning leads to a stable improvement in sensory function through repeated exposure to stimuli (Fahle, 2005; Gold & Watanabe, 2010). While most improvements in perception happen during development (Atkinson, Braddick, & Moar, 1977; Gibson, 1969), perceptual learning is still possible throughout adulthood by means of extensive training and neuronal plasticity (Fahle & Poggio, 2002). Perceptual learning results in an improved perception of stimuli. In particular, visual perceptual learning has been studied in the context of rehabilitation in clinical conditions, aging, as well as education (Dosher & Lu, 2017). Moreover, improvements through training have been shown in the auditory system (Atienza, Cantero, & Dominguez-Marin, 2002; Moore, Amitay, & Hawkey, 2003), the olfactory system (Moreno et al., 2009; Wilson & Stevenson, 2003), taste perception (Owen & Machamer, 1979), and the somatosensory system (Pleger et al., 2003; Sathian & Zangaladze, 1998).

To date, however, no demonstrations of perceptual learning have been reported in passive self-motion perception relying primarily on the vestibular organs. Hartmann and colleagues found no perceptual learning of passive self-motion for yaw rotations (semicircular canal input) and leftward-rightward translations (y-translation; otolith input) in the dark (Hartmann, Furrer, Herzog, Merfeld, & Mast, 2013). Interestingly, perceptual learning of self-motion direction occurred when participants were exposed to a visual scene during both training and testing, thus combining visual and vestibular information during both training and testing. The difference in learning outcome between the two conditions was explained by the highly multisensory nature of spatial orientation and the importance of visual information for self-motion perception (Wolfe et al., 2018).

Indeed, it has been argued that multisensory stimuli are optimal for perceptual learning (Shams & Seitz, 2008). In their review, the authors argue that unisensory stimuli can exclusively alter brain structures involved in processing this specific type of stimulus. Multisensory stimuli, however, can alter not only brain structures involved in processing unisensory inputs, but also functional connectivity between unisensory structures as well as multisensory structures. Thus, training with multisensory stimuli increases the probability and efficiency of perceptual learning. Interestingly, multisensory training increases perceptual learning even for test stimuli that are unisensory (Guo & Guo, 2005; Seitz, Kim, & Shams, 2006; Von Kriegstein & Giraud, 2006). These studies imply that a multisensory setting facilitates perceptual learning.

The goal of the present study was to investigate whether perceptual learning of passive self-motion without visual input is possible when the passive self-motion stimuli are composed of simultaneous otolith and semicircular canal information. The earlier Hartmann et al. (2013) study used motion stimuli that either activated the semicircular canals or the otoliths in isolation. A combined motion stimulus activating both the otoliths and the semicircular canals, such as roll tilt, can be considered a multisensory stimulus since it involves different vestibular sensory organs. It should be noted that self-motion perception thresholds need not depend exclusively on vestibular information. Somatosensory, proprioceptive, and visceral signals cannot be ruled out completely (Jian, Shintani, Emanuel, & Yates, 2002; Lim, Karmali, Nicoucar, & Merfeld, 2017; Mittelstaedt, 1992, 1996; Yates & Stocker, 1998). However, a comparison of self-motion thresholds in healthy subjects and bilateral vestibular patients suggests that the vestibular system plays the predominant role in self-motion perception for roll tilt (Valko, Lewis, Priesol, & Merfeld, 2012).

We investigated perceptual learning with 0.2-Hz roll tilt motion stimuli because they require the brain to combine otolith and semicircular information (Lewis, Priesol, Nicoucar, Lim, & Merfeld, 2011; Lim et al., 2017). Subjects were trained in a 0.2-Hz roll tilt direction discrimination task. To assess changes in self-motion perception, 0.2-Hz roll tilt thresholds were measured before and after training. In addition, we measured transfer of learning to a higher frequency (1 Hz) and different motion axes (pitch, y-translation). Most studies on perceptual learning report that learning is specific to the trained condition (Dosher & Lu, 2017; Parkosadze, Otto, Malania, Kezeli, & Herzog, 2008), suggesting that transfer effects are rather unlikely. However, previous findings of learning transfer from multisensory to unisensory conditions (Seitz et al., 2006) suggest possible transfer effects from roll tilt direction discrimination to y-translation direction discrimination.

## Methods

### Subjects

Thirty subjects took part in this study. Ten subjects (six female, four male, aged between 22 and 31 years) were part of the training group that received a self-motion discrimination training, as well as a pre- and post-test to measure their self-motion perception thresholds. Another 20 subjects (12 female , eight male, aged between 21 and 38 years) were tested as control subjects (divided in two different groups with *n* = 10; see *Motion stimuli* for details) who received no training, but took part in the pre- and post-test threshold measurements with the same time interval in between. Subjects indicated no history of vestibular disorders. They were compensated with cash or course credits for participating in the experiment. All subjects gave informed consent prior to the study. The study was carried out in accordance with the Declaration of Helsinki and ethical approval was obtained from the Ethics Committee of the University of Bern.

### Motion stimuli

The motion stimuli used for the training and the threshold measurements were applied using a six degrees of freedom motion platform (6DOF2000E, MOOG Inc., East Aurora, NY, USA). All stimuli consisted of single cycles of sinusoidal acceleration motion profiles (Grabherr, Nicoucar, Mast, & Merfeld, 2008) with a frequency of either 0.2 Hz or 1 Hz along different motion axes dependent on the condition. Participants were blindfolded and seated on a cushioned chair mounted on the motion platform. The head was fixated and participants were wearing headphones playing white noise to cover the sound from the motion platform.

During training the subjects of the training group experienced roll tilts about an earth horizontal axis with the center of rotation on the level of the motion platform, just below the hip. The frequency of the motion was 0.2 Hz. The peak velocity of the stimuli was determined individually for each subject based on the performance at the pretest measurement. We aimed at an accuracy of about 65% at the start of the training to maximize efficiency of the training (Hartmann et al., 2013).

At the pre- and post-test measurements, thresholds of self-motion perception for different motion conditions were measured for all participants. We were interested in the learning effect for the trained condition and whether there is a potential transfer to other types of motion axes and to a higher motion frequency. Thus, the pre- and post-test assessment of the experimental group also included *roll 1 Hz* (same motion axis, different frequency), *pitch 0.2 Hz* (different motion axis, semicircular canal and otolith combined, same frequency, center of rotation on the level of the motion platform), and *y-translation 0.2 Hz* (unisensory condition, otolith only, same frequency) threshold measurements*.* All 20 control subjects completed the pre- and post-test assessment in the *roll 0.2 Hz* and *roll 1-Hz condition*. Additionally, we measured *pitch 0.2 Hz* and *y-translation 0.2 Hz* thresholds in one half (n = 10) of the control subjects. We tested the other half of the control subjects (n = 10) with *pitch 1 Hz* to include another type of motion with higher frequency. Note that we wanted to avoid testing all the control thresholds within the same participants in order to reduce the duration of the threshold measurement sessions.

At the pre- and post-test measurements, we used seven different motion intensities for each direction (left/right for roll tilt and y-translation, forward/backward for pitch), resulting in 14 different stimuli. The peak velocities were 1.5 °/s, 0.85 °/s, 0.65 °/s, 0.4 °/s, 0.15 °/s, 0.1 °/s and 0.05 °/s for the roll and pitch stimuli and 0.06 m/s, 0.055 m/s, 0.05 m/s, 0.045 m/s, 0.04 m/s, 0.035 m/s, and 0.03 m/s for the y-translation stimuli. Each stimulus was presented ten times during this measurement, resulting in a total of 140 trials per motion condition. The peak velocities of each stimulus were chosen based on pilot testing in order to measure the whole spectrum of performance while accounting for interindividual differences between subjects. For the y-translation, the highest velocity was not chosen based on performance, but because it was the maximal possible velocity due to displacement limitations of the motion platform.

### Procedure

#### Pretest

The first appointment served the purpose of measuring the psychometric functions for all tested motion axes and frequencies. For the training group, performance in the pretest measurement of the *roll 0.2-Hz condition* additionally determined the peak velocity of the training stimuli. Subjects completed all four motion conditions in this session and the order of the conditions was counterbalanced across subjects. Prior to each motion condition, we administered 24 practice trials consisting mostly of suprathreshold stimuli to allow for familiarization with the task and motion condition. Then, during the actual threshold measurement, subjects performed the motion direction discrimination task for the respective motion condition. A sound indicated the onset of the motion. Subjects responded by button press to indicate whether they were rotated (or translated) to the left or right (or backward or forward). Each motion condition took between 20 and 40 min depending on the motion frequency and response speed. Including breaks between motion conditions, the pretest took around 3 h.

#### Training

The training group received a *roll 0.2 Hz* motion direction discrimination training starting a day after the pretest. The training was comparable to the threshold measurement in the pretest in the *roll 0.2-Hz condition*, with the difference that only one motion intensity was used. This motion intensity was chosen for each subject individually based on their fitted psychometric function in the pretest. We chose the peak velocity such that the performance accuracy (i.e., percent correct) in the first training sessions would be about 65%. If accuracy was below 55% or above 85% in the first three training blocks we adapted the difficulty of the task. The training was administered over 6 days with a 2-day break over the weekend after either the third or the fourth training day. Each day, subjects completed three blocks of the direction discrimination training with a 100 trials. Thus, the training consisted of 300 trials (90 min) per day, which was a compromise between the minimal number of trials required for perceptual learning to take place (160–400; Aberg, Tartaglia, & Herzog, 2009) and a duration that was a limited burden on subjects. Over the course of the 6 training days, subjects trained for approximately 9 h and they completed 1,800 trials. In order to maximize learning efficiency, participants received feedback in the form of a short tone when they made a mistake (De Niear, Noel, & Wallace, 2017; Fahle & Edelman, 1993; Goldhacker, Rosengarth, Plank, & Greenlee, 2014).

#### Post-test

The post-test served to assess training effects in the *roll 0.2-Hz condition* as well as in the transfer conditions. For the training as well as the control group, the post-test session took place on the ninth day after the pretest session (on the first day after the last training session for the training group). The time of measurement was kept the same as in the pretest session. For all motion conditions, subjects again received the 24 practice trials before the measurement started. For each subject, the order of the motion conditions was the same as in the pretest.

### Data analysis

Responses were analyzed using Bayesian Hierarchical Generalized Linear Models that estimate fixed effects and varying effects for each subject (partial pooling models). This has clear advantages over the more traditional method of fitting data to each subject individually (no-pooling models) and then further analyzing the estimated parameters. On a conceptual level, partial pooling models assume that there is a distribution of perceptual thresholds in the population (i.e., fixed or group-level effects), and all subjects are random draws from this distribution (i.e., varying effects). This allows for estimation of group-level means and additional varying effects for individual subjects. In no-pooling models, subjects are treated as completely independent because no assumption is made about an underlying population. For parameter estimation, partial pooling models lead to more reliable parameter estimates (Katahira, 2016). Firstly, partial pooling models induce shrinkage of the parameter estimates for the subjects (varying effects) towards the group means, which reduces overfitting of the data (Ellis, Klaus, & Mast, 2017; Gelman & Hill, 2007; Katahira, 2016). Another important advantage of partial pooling models is that uncertainty concerning the parameters of each subject is considered when estimating group-level effects. This is achieved by weighting the data of individual subjects according to the uncertainty that is associated with it. When analyzing a point estimate of the parameters for each subject individually, uncertainty is not taken into account. In that case, each estimate is weighted equally and independent of its uncertainty. Lastly, data from all subjects are used to estimate varying effects for each subject individually, thus increasing the reliability of estimates by using all possible information (Gelman & Hill, 2007). A drawback of using Bayesian Hierarchical Generalized Linear Models is the increased complexity and computational power needed for data analysis. However, modern software packages such as BRMS and RStan have made the application of such models more convenient (Bürkner, 2017; Stan Developent Team, 2018).

For the pre/post comparison, responses were analyzed using a Bayesian Hierarchical Generalized Linear Model with a probit link function. The probability of a rightward (or forward for pitch) response was predicted by peak velocity (positive = rightward/forward, negative = leftward/backward), the time (pre vs. post) and the group (training vs. control; note that the two control groups were not separated for data analysis). Dummy coding was used for categorical variables, with *pre* and *training* being the reference categories (see Table 1 for a description of model parameters). This allowed for estimation of psychometric functions before and after the training (or waiting period) for both the training and control group. Varying intercept and varying slopes for all variables (velocity, time) except group, which is a between-subjects variable, were implemented (Barr, Levy, Scheepers, & Tily, 2013). We define perceptual learning as an increase in slope of the psychometric function that reflects sensitivity (Wichmann & Hill, 2001). For comparability with other studies on self-motion perception, threshold values are also reported. Threshold values are the inverse of the slope parameter of the psychometric function and represent the velocity value at which a subject has an accuracy of 84% if there is no bias (Merfeld, 2011).

**Table 1:** Description of model parameters in the pre/post comparison

Parameter	Description
b_intercept	The probability of the response right (or forward) in the pretest for the training group when the velocity is 0 on the probit scale (i.e., the pretest bias in the reference group).
sd_intercept	The between-subjects variability of b_intercept.
b_post	Additive effect on the intercept (bias) for the post-test.
sd_post	The between-subjects variability of b_post.
b_control	Additive effect on the intercept (bias) for the control group.
b_velocity	Effect of velocity on the probability of a right (or forward) response during the pretest for the training group on the probit scale (i.e., the pretest slope of the psychometric function in the reference group). This is the inverse of the pretest threshold.
sd_velocity	The between-subjects variability of b_velocity.
b_post*control	Additive effect on the intercept (bias) for the post-test in the control group.
b_post*velocity	Additive effect on the slope of the psychometric function for the post-test in the training group. A positive value indicates higher sensitivity or lower threshold after the training. / The variability across subjects vor this parameter.
sd_post*velocity	The between-subjects variability of b_post*velocity.
b_control*velocity	Additive effect on the slope of the psychometric function for the control group in the pretest. This parameter represents the difference in sensitivity between training and control group before training.
b_post*control*velocity	Additive effect on the slope of the psychometric function for the post-test in the control group. This parameter indicates whether the pre/post difference in the control group differs from the pre/post difference in the training group.

For the training data, we predicted the probability of a rightward answer by the stimulus direction (right or left). If we adapted the stimulus intensity within the first three blocks (see above), these blocks were excluded for analysis. We used effect coding for the variable stimulus direction (right = 0.5, left = −0.5). This allows for a convenient interpretation of model parameters in terms of signal detection parameters. The negative intercept can be understood as the decision criterion. The parameter for the stimulus direction can be readily interpreted as d’, a standard signal detection sensitivity index (Knoblauch & Maloney, 2012). Again, maximal varying effects structure (varying intercept and varying slopes for direction and block) that was justified by the design was implemented in this analysis (Barr et al., 2013).

Models for both pre/post comparison and data recorded during the training were estimated using brms (Bürkner, 2017) and rstan (Stan Developent Team, 2018). Weakly informative priors were used for model estimation. For population-level parameters a normal prior (mean = 0, SD = 100) was used. For all other parameters default priors provided by brms were used, and specifically for subject-level variability a half student-t distribution was used (df = 3, mean = 0, spread = 10). Parameter estimates were obtained using Markov Chain Monte Carlo Sampling (MCMC) with four independent chains of 1,000 warm-up samples and 1,000 samples drawn from the posterior distribution, which were saved for statistical inference. To make sure that the samples of the chains converged to the same posterior distribution, chains were visually inspected and R-Hat statistics were computed. All R-Hats were below 1.02, suggesting that the chains had converged to the same posterior distribution (Gelman et al., 2013). The parameter estimates representing additive effects were evaluated using the 95% credible interval (95% CrI) based on the posterior distribution. If the 95% CrI of a parameter estimate did not include 0, it was interpreted as strong evidence for an effect (Kruschke, 2013; Nicenboim & Vasishth, 2016). A maximum-likelihood approach for the same statistical models with lme4 (Bates, Mächler, Bolker, & Walker, 2015) for parameter estimation led to the same conclusions as the Bayesian analysis reported in this paper. All data, models, and code for model estimation are freely accessible on the Open Science Framework (OSF; https://osf.io/dhtq8/).

## Results

### Pre/post comparison

In the discussion of the results for each motion condition we focus mainly on the parameters b_post*velocity and b_post*control*velocity, as these parameters reflect perceptual learning. However, if there is a relevant finding (e.g., concerning the bias) it will also be discussed. The parameter b_velocity was positive in all motion conditions used in the experiment. This is not surprising, as it suggests that in the reference category subjects were able to perform the task and that their discrimination ability improved with increasing stimulus level (i.e., their psychometric function showed a positive slope).

#### Roll 0.2 Hz

**Fig. 1:**
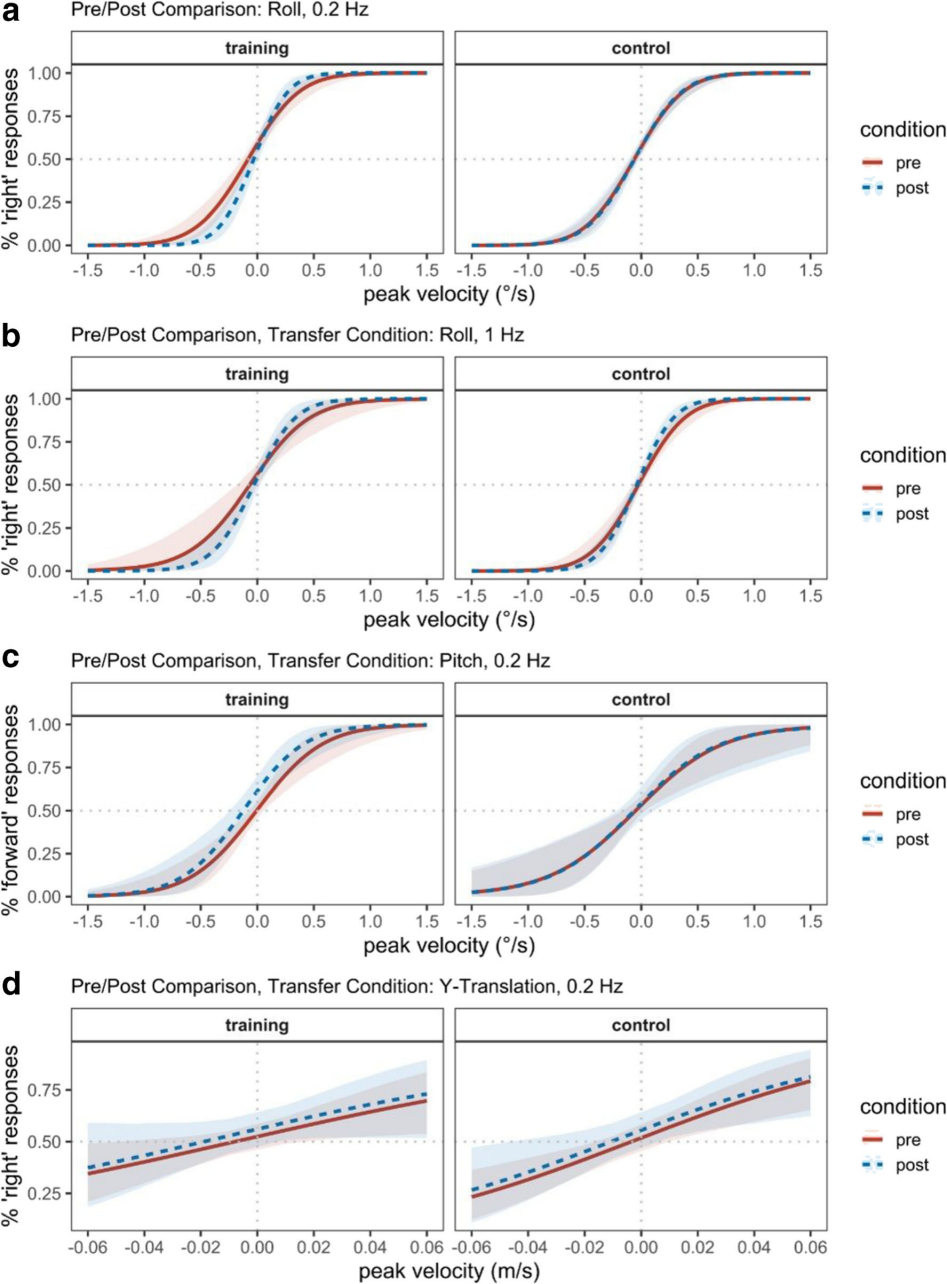
Visualization of fitted psychometric functions estimated with the hierarchical model. **a** Proportion of right responses as a function of angular velocity in the roll 0.2-Hz condition. There is an increase in slope of the psychometric function (i.e., increased discriminability) between the two measurements in the training group (left panel), but not in the control group (right panel). **b** Proportion of right responses as a function of angular velocity in the roll 1-Hz condition. The slope of the psychometric function in the post-test is increased compared to the pretest for the training group (left panel) and for the control group (right panel). **c** Proportion of forward responses as a function of angular velocity in the pitch 0.2-Hz condition. There is neither an increase in slope for the training group (left panel) nor for the control group (right panel). **d** Proportion of right responses as a function of velocity in the y-translation 0.2-Hz condition. There is neither an increase in slope for the training group (left panel) nor for the control group (right panel)

**Fig. 2:**
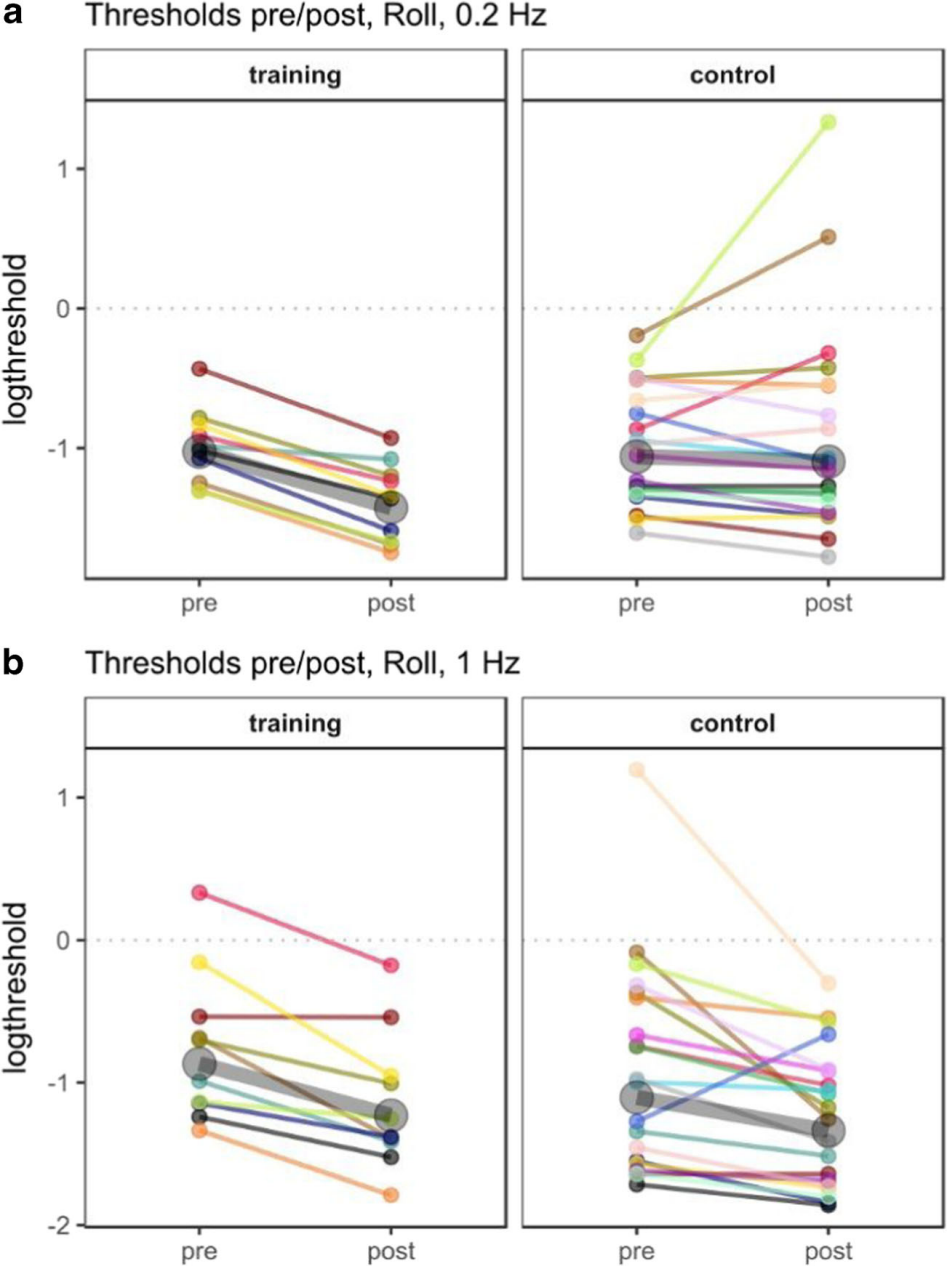
Perceptual thresholds for all subjects in the roll 0.2-Hz (**a**) and roll 1-Hz (**b**) motion conditions. Data points represent varying effects of logthresholds for each subject, which were estimated in the hierarchical generalized linear model. Each color represents a single subject before and after the training or the waiting period. The training group is visualized in the left panels, and the control group in the right panels. Larger gray circles represent population estimates of logthresholds. Thresholds were log transformed for better scaling of the visualization

A full account of all parameter estimates for the *roll 0.2 Hz* motion condition can be found in Table 2 and is illustrated in Fig. 1a. There was an increase in the slope of the psychometric function (i.e., decrease in threshold) when comparing the pre- and post-test condition in the training group (b_post*velocity = 1.37, 95% CrI [0.61; 2.18]). The negative three-way interaction of velocity, time of measurement, and group indicates that this increase in sensitivity was not present in the control group (b_post*control*velocity = -1.26, 95% CrI [-2.18; -0.35]). Indeed, supplementary analysis with the control group as reference confirms that there is no improvement between pre- and post-test evident in the control group (b_post*velocity = 0.22, 95% CrI [-0.40; 0.66]; see Table S1 in the Supplementary Materials for a detailed account of parameter estimates of the model with the control group as reference). Training improved sensitivity in 0.2-Hz roll tilt discrimination. This is evidence for perceptual learning of self-motion discrimination in the dark. In the training group, the average threshold across subjects was reduced 33% from 0.36 °/s (range: 0.27–0.65 °/s) before training to 0.24°/s (range: 0.17–0.40 °/s) after the training. Moreover, each individual subject showed a reduction in threshold between the two measurements (see Fig. 2a). In the control group, the threshold was 0.35 °/s (range: 0.20–0.82 °/s) at the first measurement and 0.33°/s (range: 0.17–3.80 °/s) at the second measurement.

**Table 2:** Model summary for the roll 0.2 Hz pre/post comparison

Parameter	Estimate	SD	95% CrI	Eff. Sample
b_intercept	0.23	0.07	[0.09; 0.36]	2,131
b_post	-0.09	0.09	[-0.27; 0.08]	1,953
b_control	-0.05	0.08	[-0.21; 0.12]	2,105
b_velocity	2.78	0.40	[1.98; 3.59]	1,579
b_post*control	0.09	0.11	[-0.11; 0.30]	1,974
b_post*velocity	1.37	0.40	[0.61; 2.18]	2,184
b_control*velocity	0.10	0.50	[-0.86; 1.09]	1,637
b_post*control*velocity	-1.26	0.46	[-2.18; -0.35]	2,346
sd_intercept	0.16	0.04	[0.07; 0.25]	1,534
sd_post	0.17	0.06	[0.04; 0.30]	895
sd_velocity	1.18	0.22	[0.82; 1.69]	1,293
sd_post*velocity	0.82	0.22	[0.43; 1.31]	1,828

*Notes.* 95% CrI *=* 95% credible interval. Eff. Sample = effective sample size. Population-level parameters are highlighted if the credible interval does not contain 0

Additionally, the model also suggests an overall bias toward the response right for the reference group (b_intercept = 0.23, 95% CrI [0.09; 0.36]). A lack of any other effects concerning the response tendencies suggests that there is a preference for rightward responses in all motion conditions. This is reflected in the slight leftward shift of all psychometric functions in Fig. 1a.

#### Roll 1 Hz

All parameter estimates for the *roll 1-Hz condition* are summarized in Table 3, and Fig. 1b shows the psychometric functions. We found a similar increase in slope of the psychometric function as in the trained motion condition (*Roll 0.2 Hz*) when comparing the pre- and post-test condition in the training group (b_post*velocity = 1.05, 95% CrI [0.24; 1.93]). We found no three-way interaction between velocity, time of measurement, and group, suggesting that the change between the first and the second measurement was the same for the control group (b_post*control*velocity = -0.26, 95% CrI [-1.36; 0.77]). This implies that there was also an increase in slope for the control group in the *roll 1 Hz* motion condition. This is supported by supplementary analysis with the control group as reference category, which shows that the slope of the psychometric function of the control group increased between pre- and post-test (b_post*velocity = 0.78, 95% CrI [0.09; 1.47]); see Table S2 in the Supplementary Materials for a detailed account of parameters of the model with the control group as reference. The mean thresholds before and after training were 0.42 °/s (range: 0.26–1.39 °/s) and 0.29 °/s (range: 0.17–0.84 °/s), respectively, in the training group. This is a reduction of 31%. In the control group, the th;eshold was 0.33 °/s (range: 0.18–3.30 °/s) at the first measurement and 0.26 °/s (range: 0.16–0.74 °/s) at the second measurement, thus, it was reduced by 21% even without training. Just like in Roll 0.2 Hz, each subject of the training group showed a reduced threshold after training (Fig. 2b). In the control group, thresholds were reduced in all but one subject.

**Table 3:** Model summary for the roll 1 Hz pre/post comparison

Parameter	Estimate	SD	95% CrI	Eff. Sample
b_intercept	0.16	0.08	[-0.01; 0.32]	1,751
b_post	-0.06	0.10	[-0.25; 0.15]	1,575
b_control	-0.08	0.10	[-0.28; 0.11]	1,791
b_velocity	2.38	0.56	[1.29; 3.54]	1,600
b_post*control	0.13	0.12	[-0.11; 0.37]	1,884
b_post*velocity	1.05	0.43	[0.24; 1.93]	2,656
b_control*velocity	0.64	0.70	[-0.76; 1.95]	1,554
b_post*control*velocity	-0.26	0.54	[-1.36; 0.77]	2,486
sd_intercept	0.22	0.05	[0.14; 0.32]	1,524
sd_post	0.24	0.06	[0.13; 0.37]	1,352
sd_velocity	1.69	0.28	[1.23; 2.32]	1,693
sd_post*velocity	1.11	0.27	[0.65; 1.72]	1,893

*Notes.* 95% CrI *=* 95% credible interval. Eff. Sample = effective sample size. Population-level parameters are highlighted if the credible interval does not contain 0

#### Pitch 0.2 Hz

All parameter estimates for the *pitch 0.2-Hz* condition can be found in Table 4 and psychometric functions are visualized in Fig. 1c. We found no increase in slope of the psychometric function for the *pitch 0.2-Hz* condition between pre- and post-test in the training group (b_post*velocity = 0.20, 95% CrI [-0.36; 0.74]). There was also no three-way interaction between velocity, time of measurement and group (b_post*control*velocity = -0.13, 95% CrI [-0.87; 0.60]). Neither the experimental group nor the control group improved between the pre- and post-test measurement. In terms of thresholds, the training group had a mean threshold of 0.47 °/s (range: 0.27–0.89 °/s) before training and 0.43 °/s (range: 0.23–0.85 °/s) after training. In the control group, the threshold was 0.61 °/s (range: 0.23–1.86 °/s) at the first measurement and 0.59 °/s (range: 0.21–1.80 °/s) at the second measurement.

**Table 4:** Model summary for the pitch 0.2 Hz pre/post comparison

Parameter	Estimate	SD	95% CrI	Eff. Sample
b_intercept	0.01	0.07	[-0.12; 0.14]	2,311
b_post	0.28	0.12	[0.05; 0.51]	1,549
b_control	0.07	0.09	[-0.12; 0.26]	2,094
b_velocity	2.15	0.46	[1.25; 3.09]	1,228
b_post*control	-0.25	0.16	[-0.58; 0.06]	1,689
b_post*velocity	0.20	0.27	[-0.36; 0.74]	1,884
b_control*velocity	-0.51	0.65	[-1.80; 0.78]	1,187
b_post*control*velocity	-0.13	0.36	[-0.87; 0.60]	1,818
sd_intercept	0.16	0.05	[0.07; 0.28]	1,853
sd_post	0.29	0.08	[0.15; 0.48]	1,580
sd_velocity	1.40	0.30	[0.93; 2.12]	1,101
sd_post*velocity	0.62	0.20	[0.27; 1.06]	1,803

*Notes.* 95% CrI *=* 95% credible interval. Eff. Sample = effective sample size. Population-level parameters are highlighted if the credible interval does not contain 0

The parameter b_post suggests that there was a bias favoring forward responses in the reference group after the training session that was not present at the first measurement (b_post = 0.28, 95% CrI [0.05; 0.51]). This suggests that there was a shift in the decision criterion caused by the training.

#### Y-translation 0.2 Hz

Parameter estimates in the *y-translation 0.2-Hz condition* are summarized in Table 5 and visualized in Fig. 1d. The slope of the psychometric function in the *y-translation 0.2-Hz condition* between pre- and post-test in the training group did not increase (b_post*velocity = 0.32, 95% CrI [-5.12; 6.01]). We found no three-way interaction between velocity, time of measurement, and group (b_post*control*velocity = -0.13, 95% CrI [-0.87; 0.59]), thus there was no improvement in the control group either. In the training group, the mean threshold was 0.13 m/s (range: 0.08–0.46 m/s) before training and 0.12 m/s (range: 0.03–15.07 m/s) after the training. In the control group, the threshold was 0.08 m/s (range: 0.02–0.52 m/s) at the first measurement and 0.08 m/s (range: 0.01–0.41 m/s) at the second measurement.

**Table 5:** Model summary for the Y-translation 0.2 Hz pre/post comparison

Parameter	Estimate	SD	95% CrI	Eff. sample
b_intercept	0.06	0.07	[-0.08; 0.21]	1,823
b_post	0.09	0.08	[-0.06; 0.24]	2,056
b_control	-0.02	0.10	[-0.22; 0.19]	1,561
b_velocity	7.80	3.23	[1.41; 14.46]	1,499
b_post*control	0.00	0.11	[-0.22; 0.24]	1,983
b_post*velocity	0.32	2.85	[-5.12; 6.01]	2,251
b_control*velocity	5.38	4.56	[-3.55; 14.49]	1,738
b_post*control*velocity	-0.30	4.18	[-8.51; 7.97]	2,219
sd_intercept	0.20	0.05	[0.12; 0.31]	1,976
sd_post	0.18	0.07	[0.04; 0.32]	1,155
sd_velocity	9.98	2.08	[6.69; 14.78]	1,977
sd_post*velocity	8.44	1.99	[5.24; 13.00]	2,418

*Notes.* 95% CrI *=* 95% credible interval. Eff. Sample = effective sample size. Population-level parameters are highlighted if the credible interval does not contain 0

#### Pitch 1 Hz

One subject had to be excluded in this condition due to a mistake in data recording. No increase in sensitivity between the first and second measurement (b_velocity*post = -0.04, 95% CrI [-1.46; 1.37]) was found (summary of results in Table 6) in the subjects tested with *pitch 1 Hz*. The threshold for this group was 0.36 °/s (range: 0.22–1.27 °/s) at the first measurement and 0.37 °/s (range: 0.19–1.43 °/s) at the second measurement.

**Table 6:** Model summary for the pitch 1 Hz pre/post comparison

Parameter	Estimate	SD	95% CrI	Eff. sample
b_intercept	0.09	0.11	[-0.14; 0.32]	1,744
b_post	-0.02	0.15	[-0.32; 0.29]	1,928
b_velocity	2.76	0.63	[1.56; 4.04]	2,136
b_post*velocity	-0.04	0.70	[-1.46; 1.37]	2,225
sd_intercept	0.28	0.12	[0.12; 0.57]	2,575
sd_post	0.38	0.17	[0.14; 0.77]	2,328
sd_velocity	1.69	0.64	[0.85; 3.28]	1,835
sd_post*velocity	1.77	0.80	[0.65; 3.79]	1,883

*Notes.* 95% CrI *=* 95% credible interval. Eff. Sample = effective sample size. Population-level parameters are highlighted if the credible interval does not contain 0

### Training effect

**Fig. 3:**
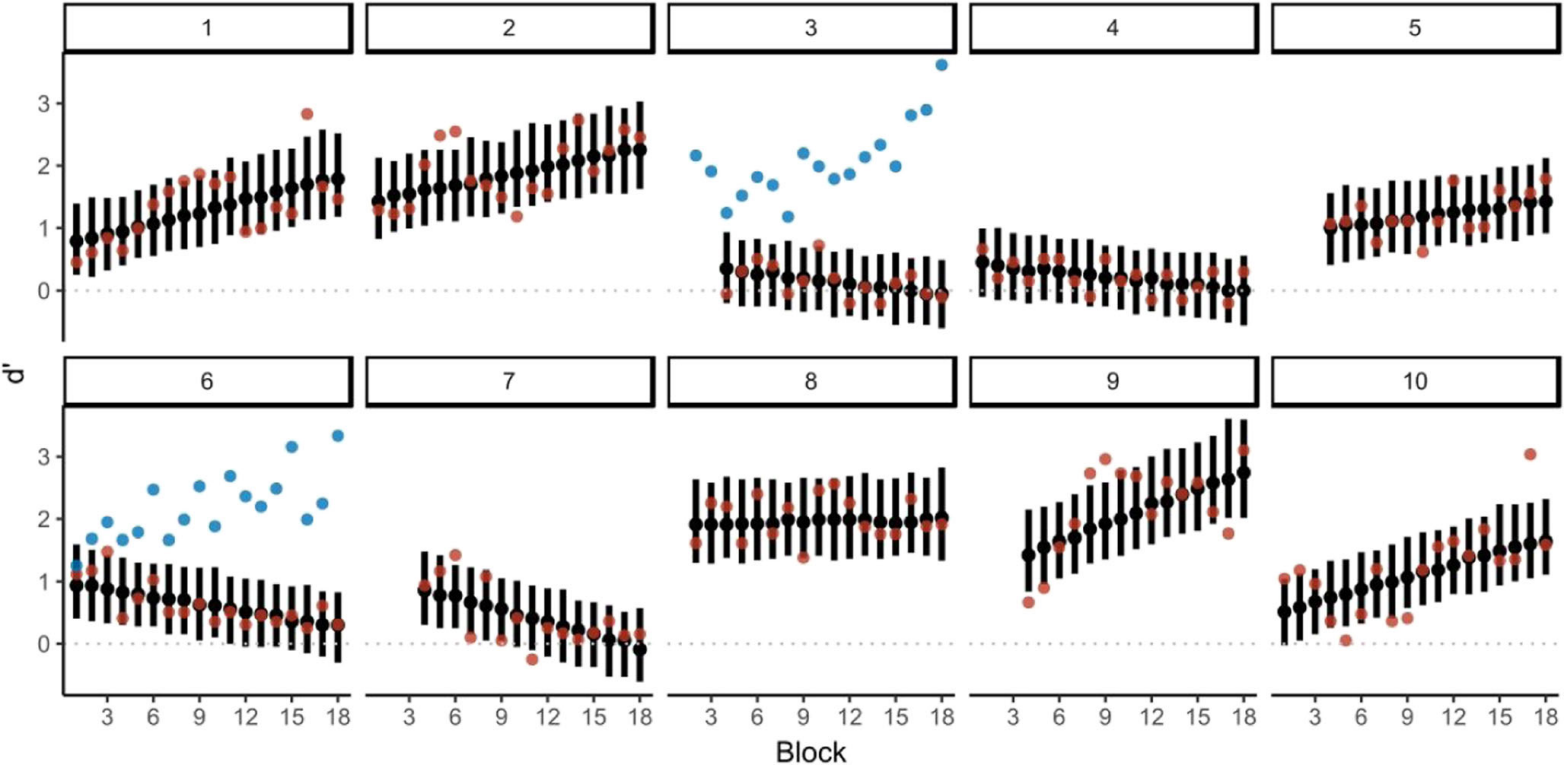
Visualisation of d’ over the training blocks for each subject individually (random effects). Black dots indicate model prediction of d’ for each block with 95% CrI. Red dots are d’ that were calculated on the basis of the proportions of hits and false alarms for each subject and block. The blue dots (subjects 3 and 6) represent d’ estimated on the basis of the proportions of hits and false alarms for the second time they completed the training. These data were not included in the fitted model, and only serve to illustrate the hypothesized explanation that stimuli were too difficult in the training sessions. A lack of data points in the first three sessions indicates that for this subject the motion intensity was changed and the data before the change of motion intensity was not included in the analysis

The effects of training vary between individuals. d’ for each subject (varying effects) during training show that some subjects improved over time. Five out of the ten subjects have a positive slope of d’ over the course of the 18 blocks. For the remaining five subjects, one shows a zero slope, and four show a negative slope of d’ over the 18 training blocks. See Table 7 and Fig. 3 for a detailed summary of the varying effects between subjects. Two of the subjects with a negative slope were asked to repeat the training with a slightly higher velocity, to test whether training stimuli were too difficult for learning to be evident. Indeed, visual inspection indicates a positive learning curve with the easier stimuli (see blue dots in Fig. 3).

**Table 7:** Summary of effects of the slope of d’ as a function of the block for each subject (varying effects)

Subject	Estimate	SD	95% CrI
1	0.06	0.01	[0.03; 0.08]
2	0.05	0.01	[0.02; 0.07]
3	-0.03	0.01	[-0.06; -0.00]
4	-0.02	0.01	[-0.05; -0.00]
5	0.03	0.01	[0.00; 0.06]
6	-0.04	0.01	[-0.06; -0.02]
7	-0.07	0.01	[-0.09; -0.04]
8	0.01	0.01	[-0.02; 0.03]
9	0.09	0.02	[0.06; 0.12]
10	0.07	0.01	[0.04; 0.09]

*Note:* 95% CrI *=* 95% credible interval.N

**Fig. 4:**
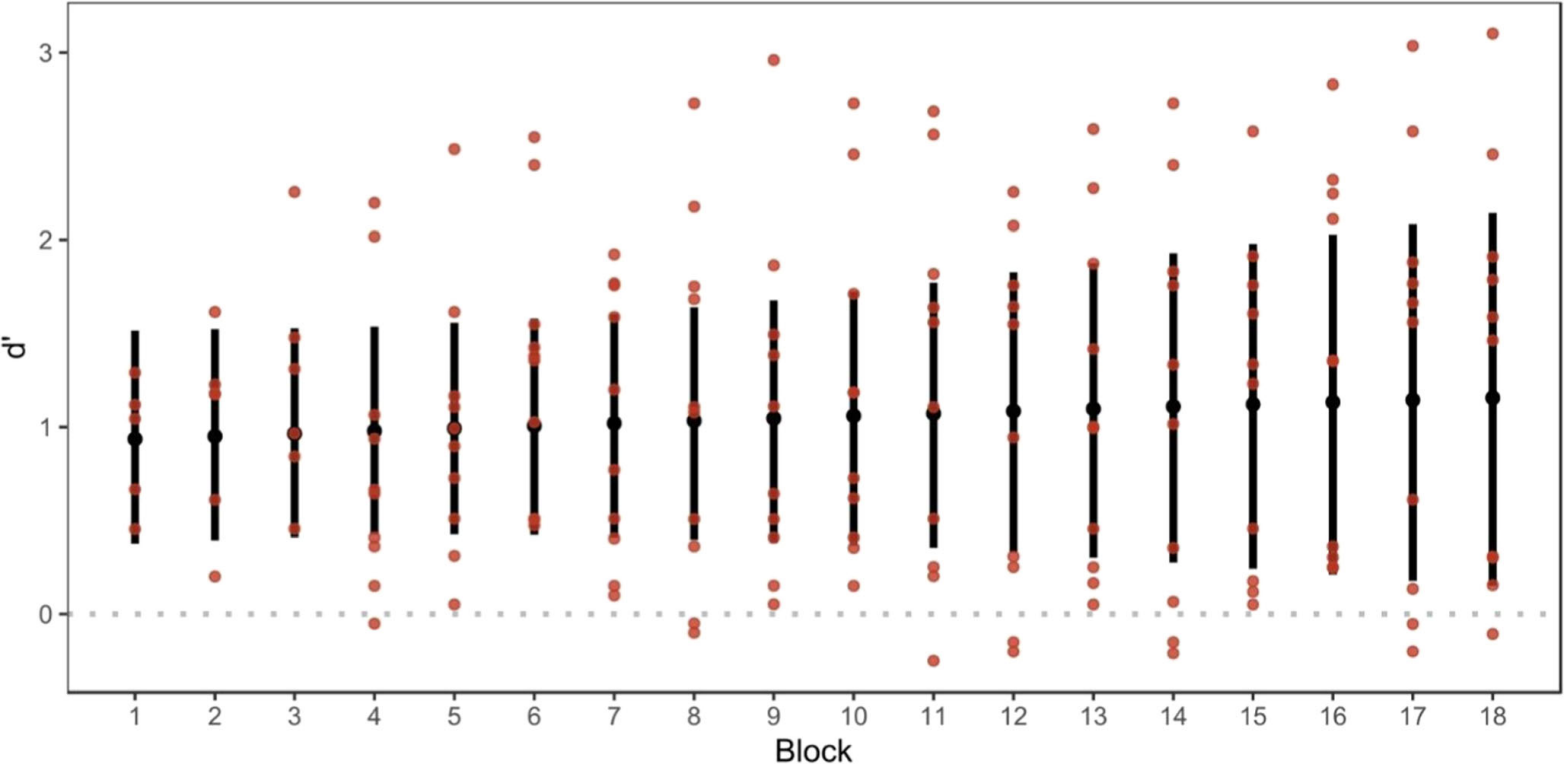
Model fit of d’ as a function of the block. Black dots are mean population estimates of d’ with the bars indicating the 95% CrI. Red dots are d’, which were calculated on the basis of the proportions of hits and false alarms for each subject and block

On the population level, d’ in the first block of training is significantly higher than 0 (b_direction = 0.94, 95% CrI [0.51; 1.38]; sd_direction = 0.64, 95% CrI [0.35; 1.18]). A d’ of 0 corresponds to chance performance in a discrimination task. Thus, participants were above chance in discriminating leftward from rightward rotations in the first block. The additive effect of the block on d’, i.e. the slope of d’ over the blocks, did not differ from 0 (b_direction*block = 0.01, 95% CrI [-0.03; 0.06]; sd_direction*block = 0.06, 95% CrI [0.04; 0.11]) on the population level. This suggests that on the population level, d’ did not increase as a function of the block number and performance stayed the same during the training task over the course of the 18 training blocks. A visualisation of d’ as a function of the block for the population is shown in Fig. 4.

Analysis of the response tendency did not reveal any substantial biases on the population level. The intercept of the modelled training data does not differ from 0, suggesting that participants did not have a bias for either leftward or rightward responses in the first block of training (b_Intercept = 0.06, 95% CrI [-0.12; 0.25]; sd_Intercept = 0.28, 95% CrI [0.17; 0.48]). The additive effect of the block number on the Intercept, i.e. the slope of the bias over the block number, does not differ from 0 (b_block = 0.00, 95% CrI [-0.01; 0.02]; sd_block = 0.03, 95% CrI [0.02; 0.05]). This suggests that the bias did not change over the course of the training.

## Discussion

Subjects were trained in a roll tilt 0.2-Hz self-motion direction discrimination task in the dark. Self-motion perception thresholds for different motion frequencies and axes were assessed before and after training. After 6 days of training (9 h, 1,800 trials), perceptual thresholds were reduced by 33% in the 0.2-Hz roll direction discrimination task. This indicates better roll direction discrimation after the training.

This is – to our knowledge – the first demonstration of perceptual learning of self-motion perception in the dark. In a previous study, Hartmann et al. (2013) used yaw rotations about an earth vertical axis (semicircular canal input only) and y-translations (otolith input only) and found no evidence for perceptual learning in the dark. Here, we used 0.2-Hz roll tilts (combined otolith and semicircular canal input) about an earth horizontal axis and we found learning. In roll tilt perception, the dynamic signal from the semicircular canals is integrated with gravitational cues from the otoliths (Lim et al., 2017). We conclude that the integration of these signals is most likely responsible for perceptual learning. This is in line with two recent studies on learning of dynamic balancing (Vimal, DiZio, & Lackner, 2017; Vimal, Lackner, & DiZio, 2018). In these studies, subjects were able to learn a dynamic balancing task in an upright roll rotation task or in a supine yaw rotation task. Removing the gravitational cues in the supine roll rotation task and the upright yaw rotation task impaired overall performance and learning of the balancing task.

We did not find any transfer of perceptual learning to the pitch 0.2-Hz or the y-translation 0.2-Hz condition. This lack of transfer is in line with most perceptual learning studies and the previous study on perceptual learning of self-motion perception (Dosher & Lu, 2017; Hartmann et al., 2013). A transfer to the unisensory y-translation condition could have been expected based on the studies on multisensory perceptual learning (Shams & Seitz, 2008). However, it should be noted that the otolith input during y-translation is purely due to linear translation and thus is not directly comparable to gravitational cues present in the roll tilt.

The perceptual improvement appears to be specific to the roll plane. However, we did observe increased performance after 0.2-Hz roll tilt training in the roll tilt 1-Hz condition. This may not represent a transfer effect because we found a similar increase in sensitivity in the 1-Hz condition for the control group that received no training. Fast learning has been reported in visual tasks (Fahle, Edelman, & Poggio, 1995; Poggio, Fahle, & Edelman, 1992) and may account for the improvement in the 1-Hz condition without any training. Control subjects in our study completed a total of 280 trials, which is in line with the number of trials used in studies on fast perceptual learning. However, more research is needed to replicate and further investigate the unexpected finding obtained with 1-Hz roll motion.

Given that we found increased performance after the training in the roll 0.2-Hz condition on the population level, it may seem surprising that we did not find a corresponding increase in d’ during the training. When looking at d’ as a function of the training block, we found that d’ remains unchanged during the training on the population level. Looking at effects for each subject separately, we can see that half the subjects show a positive association between block and d’ as would be expected when perceptual learning occurs. In the subjects who showed a negative association, d’ was relatively close to zero during most blocks. This implies that the chosen velocity was too difficult for subjects and performance was close to chance, which impairs learning and makes measurements noisier. Thus, even if there was learning, it might not be visible in d’. Simulation studies show that even in the absence of human variation, threshold assays vary about 10–20% depending on the number of simulated trials (Chaudhuri & Merfeld, 2013). Indeed, repeated threshold measurements in humans show variations in thresholds consistent with simulations (Clark, Galvan-Garza, Bermudez Rey, Yi, & Merfeld, 2015; Clark & Merfeld, 2016). Given these findings, it is likely that we underestimated the thresholds of these subjects and, thus, the motion intensity during training was too difficult for learning to be evident during training (Goldhacker et al., 2014). Indeed, when repeating the training in two subjects with a higher velocity, d’ appears to increase over time (blue dots in Fig. 3). It is important to point out that the training did lead to overall improved sensitivity as assessed by the pre- and post-test findings – suggesting that there was a training effect despite the observed absence of changes in d’ during training.

Perceptual learning of self-motion perception can be of importance for rehabilitation in the context of vestibular disease and for prevention of falls in the context of reduced vestibular function associated with aging (Agrawal, Carey, Della Santina, Schubert, & Minor, 2009; Agrawal et al., 2012; Allen, Ribeiro, Arshad, & Seemungal, 2017). Heightened self-motion perception thresholds that are thought to be caused by reduced vestibular function due to age have been demonstrated (Agrawal, Bremova, Kremmyda, Strupp, & MacNeilage, 2013; Bermúdez Rey et al., 2016; Bremova et al., 2016; Iwasaki & Yamasoba, 2015; Kingma, 2005; Roditi & Crane, 2012). A recent study showed that decreased balance test performance is associated with higher age and increased self-motion perception thresholds especially in a roll 0.2-Hz condition (Karmali, Bermúdez Rey, Clark, Wang, & Merfeld, 2017). A re-analysis of these data showed that 50% of the age-related balance decline found in this data set was mediated by the aforementioned increase in 0.2-Hz roll tilt thresholds (Beylergil, Karmali, Wang, Bermúdez Rey, & Merfeld, 2019). Thus, reducing roll tilt 0.2-Hz direction discrimination thresholds may eventually prove to be a useful intervention to improve balance and reduce falls in elderly people. Future studies are needed to test whether the decrease in perceptual thresholds of roll self-motion due to training reported herein can improve balance test performance, as is suggested by the correlation reported in the literature. Worse performance in balance tests is associated with higher morbidity, partly due to a risk of falling (Bermúdez Rey et al., 2016). Thus, we suggest that self-motion perception training should be further investigated with respect to potential therapeutic value.

## Conclusion

Roll tilt self-motion perceptual thresholds in the dark were decreased after 6 days of training. In other words, roll tilt direction discrimination improved. Given the previously reported correlation between roll tilt thresholds and balance, it is likely that roll tilt direction discrimination training can be beneficial for people recovering from vestibular disease and for the elderly to counteract the decrease of vestibular function due to ageing. Future studies are needed to investigate whether this increase in sensitivity causally influences balance, and thus reduces falls.

## Electronic supplementary material

ESM 1 (DOCX 28.7 kb)
