# Supplementary material for: Roll tilt self-motion direction discrimination training: First evidence for perceptual learning
Source: Atten Percept Psychophys. 2020 Jan 2;82(4):1987–99. doi: 10.3758/s13414-019-01967-2 (PMC7297830; doi:10.3758/s13414-019-01967-2)
Supplement: Supplementary file 1 — (DOCX 28.7 kb) [file 13414_2019_1967_MOESM1_ESM.docx]

Roll tilt self-motion direction discrimination training: First evidence for perceptual learning

(Supplementary Materials)

Table S1:

*Model Summary for the Roll 0.2 Hz Pre/Post Comparison with the control group as reference*

| **Parameter** | **Estimate** | **SD** | **95%CrI** | **Eff. Sample** |
| --- | --- | --- | --- | --- |
| **b_intercept** | **0.18** | **0.05** | **[0.08; 0.27]** | **2303** |
| b_post | -0.00 | 0.06 | [-0.12; 0.12] | 2093 |
| b_training | 0.05 | 0.08 | [-0.11; 0.21] | 2108 |
| **b_velocity** | **2.89** | **0.30** | **[2.32; 3.51]** | **1436** |
| b_post*training | -0.09 | 0.11 | [-0.30; 0.12] | 2362 |
| b_post*velocity | 0.11 | 0.27 | [-0.40; 0.66] | 2195 |
| b_training*velocity | -0.12 | 0.50 | [-1.13; 0.83] | 1261 |
| **b_post*training*velocity** | **1.23** | **0.46** | **[0.35; 2.15]** | **2431** |
| sd_intercept | 0.16 | 0.04 | [0.08; 0.25] | 1776 |
| sd_post | 0.17 | 0.06 | [0.05; 0.29] | 721 |
| sd_velocity | 1.18 | 0.22 | [0.81; 1.67] | 1570 |
| sd_post*velocity | 0.81 | 0.22 | [0.44; 1.29] | 2163 |

*Notes. Eff. Sample = Effective Sample Size. Population-level parameters are highlighted if the credible interval does not contain 0.*

Table S2:

*Model Summary for the Roll 1 Hz Pre/Post Comparison with the control group as reference*

| **Parameter** | **Estimate** | **SD** | **95%CrI** | **Eff. Sample** |
| --- | --- | --- | --- | --- |
| b_intercept | 0.08 | 0.06 | [-0.04; 0.19] | 1653 |
| b_post | 0.07 | 0.07 | [-0.07; 0.21] | 1973 |
| b_training | 0.09 | 0.10 | [-0.11; 0.29] | 1377 |
| **b_velocity** | **3.04** | **0.44** | **[2.18; 3.95]** | **1089** |
| b_post*training | -0.13 | 0.12 | [-0.37; 0.11] | 1889 |
| **b_post*velocity** | **0.78** | **0.35** | **[0.09; 1.47]** | **2293** |
| b_training*velocity | -0.67 | 0.73 | [-2.10; 0.76] | 934 |
| b_post*training*velocity | 0.30 | 0.55 | [-0.75; 1.41] | 2160 |
| sd_intercept | 0.22 | 0.05 | [0.14; 0.32] | 1509 |
| sd_post | 0.24 | 0.06 | [0.13; 0.37] | 1048 |
| sd_velocity | 1.72 | 0.30 | [1.22; 2.38] | 1471 |
| sd_post*velocity | 1.13 | 0.27 | [0.68; 1.71] | 1735 |

*Notes. Population-level parameters are highlighted if the credible interval does not contain 0.*
